# The utility of 24-h electrocardiogram recordings for the prediction of a sufficient number of premature ventricular complexes and mapping strategy during catheter ablation

**DOI:** 10.3389/fcvm.2025.1558130

**Published:** 2025-03-05

**Authors:** Stine Aagaard-Nilsen, Lars Andreas Dejgaard, Ole-Gunnar Anfinsen, Erik Lyseggen, Torbjørn Holm, Trine S. Fink, Hans Henrik Odland, Knut Sevre, Erik Kongsgård, Finn Hegbom, Mathis Korseberg Stokke

**Affiliations:** ^1^ Institute for Experimental Medical Research, Oslo University Hospital and University of Oslo, Oslo, Norway; ^2^ KG Jebsen Centre for Cardiac Research, University of Oslo, Oslo, Norway; ^3^ Department of Cardiology, Oslo University Hospital Rikshospitalet, Oslo, Norway

**Keywords:** arrhythmia, premature ventricular complexes, ambulatory electrocardiography, catheter ablation, outcome prediction

## Abstract

**Background and aims:**

An insufficient number of premature ventricular complexes (PVCs) during catheter ablation (CA) may prohibit adequate mapping of the site of origin. Parameters to predict this situation have not been established. Our objective was to quantify the association between preprocedural information and the probability of a sufficient number of PVCs for adequate mapping and successful CA.

**Methods:**

Clinical characteristics and results from examinations and procedural data were collected retrospectively from health journals for patients admitted for CA of PVCs from 2011 to 2020.

**Results:**

In total, 46 of 332 patients (14%) had an insufficient number of PVCs to enable adequate electroanatomical mapping. Patients with a sufficient number of PVCs had nominally more PVCs in the 24-h electrocardiogram (ECG), with a strong statistical trend [16,007 (6,509–26,205) vs. 8,332 (3,066–20,974), *p* = 0.055]. The receiver operator curve for a sufficient number of PVCs in 24-h ECGs had an area under the curve of 0.610 (95% CI 0.498–0.722, *p* = 0.055). The best predictive values were found at >10,000 PVCs per 24-h, with a positive predictive value of 67% and a negative predictive value of 57%. Patients for whom activation mapping was used as the sole mapping method had more PVCs in the 24-h ECG than did patients for whom pace mapping was added or used as an alternative [19,769 (10,564–30,526) vs. 15,237 (6,000–25,033), *p* = 0.022]. Neither acute outcome nor procedure time depended on the mapping strategy.

**Conclusion:**

The number of PVCs in a 24-h ECG was moderately associated with the presence of a sufficient number of PVCs to perform electroanatomical mapping during CA. The presence of more PVCs in the preprocedural 24-h ECG was associated with the use of activation mapping as the sole mapping strategy.

## 1 Introduction

Catheter ablation (CA) is recommended as the first-line therapy for symptomatic idiopathic premature ventricular complexes (PVCs) that originate from the right ventricular outflow tract or the left fascicles (1) and is frequently used for PVCs with other sites of origin (2). Although the success rate for CA of PVCs is >80% in most studies (2–7), the increasing demand for this resource-intensive procedure necessitates the evidence-based selection of patients who are most likely to benefit to ensure that healthcare is cost-effective. The use of robust preprocedural predictors of success is essential to guide patient selection and shared decision-making, but such predictors remain to be established.

Successful CA of PVCs relies on electroanatomical mapping of the site of origin (8), but the presence of an insufficient number of PVCs to perform such mapping at the time of CA is a challenge (9, 10). Based on previous publications, this problem occurs in 7–8% of procedures (9, 10). What constitutes a sufficient number of PVCs is partly operator-dependent, but it is generally accepted that activation mapping (AM), which is the preferred strategy, requires more PVCs than pace mapping (PM). Few studies have aimed to identify preprocedural parameters that can be used to predict whether a sufficient number of PVCs to perform CA will be present at the time of the procedure; none have identified predictors regarding which mapping strategy will be feasible. Even for the most often used parameter, i.e., PVC burden during long-term electrocardiogram (ECG) recording, the predictive value is uncertain (11, 12).

Our objective was to quantify the value of common clinical parameters as predictors of a sufficient number of PVCs and the mapping strategy used during CA, with a special emphasis on 24-h ECG. Therefore, we retrospectively analyzed data from all adult patients who had been admitted to our tertiary referral center for CA of PVCs between 2011 and 2020.

## 2 Materials and methods

### 2.1 Patient population

This study was approved by the Regional Ethical Committee of South-Eastern Norway (ID 243695) and the Data Protection Office at Oslo University Hospital. All patients were given the opportunity to prohibit the use of data from their medical records. We identified patients who had undergone invasive electrophysiology and CA procedures to treat PVCs at Oslo University Hospital Rikshospitalet from 2011 to 2020 based on a manual search among all the procedures that were labeled as “PVC” or “ventricular arrhythmias” in our hospital registry. The indication for each procedure was confirmed from the patients’ medical records. Only procedures that had been performed with PVC as the main indication were included. All data were collected from the electronic patient records.

### 2.2 Data collected from preprocedural evaluations of patients

#### 2.2.1 General patient characteristics

Data regarding age, sex, comorbidities, medication, and symptoms at the time of the procedure were collected from the electronic records of all patients.

#### 2.2.2 ECG

PVC morphology was categorized from the last available 12-lead surface ECG that had been recorded prior to the invasive procedure outside the electrophysiology lab, at rest in the supine position, and that showed at least one PVC. PVC morphology was defined as left or right bundle branch block based on the dominant deflection of the QRS complex in lead V1 (13). The superior or inferior axis was determined by the dominant vector of the QRS in leads II, III, and aVF (14). Coupling intervals were noted as the number of milliseconds from the R-peak in sinus rhythm to the R-peak of the PVC (15).

We included long-term, three-lead ECG recordings, which had been performed either as ambulatory ECG recordings for a minimum of 24 h or as in-hospital telemetric ECG surveillance with the possibility of a quantification of PVCs. The date of the 24-h ECG recording was defined as the date when the recording started. If the recording lasted for more than 24 h, the average number of PVCs per 24 h was calculated. If several recordings were available, the last recording prior to CA was used. Information concerning the use of anti-arrhythmic drugs during the 24-h ECG was not available.

We included exercise testing with continuous ECG monitoring, which was performed prior to CA. Whether or not PVCs were present during the exercise test, regardless of time point, was noted as PVCs in relation to exercise testing.

#### 2.2.3 Transthoracic echocardiograms and magnetic resonance imaging

Results from analysis of transthoracic echocardiograms were collected from descriptions in the electronic patient records. A normal ejection fraction (EF) was defined as an EF >50% for both men and women. Valvular disease was considered significant when reported as moderate or severe.

Results extrapolated from cardiac magnetic resonance imaging (MRI) were collected from descriptions in the electronic records. The presence of myocardial pathology, including fibrosis, was noted.

Abnormalities that had been identified by echocardiography or MRI were categorized as structural heart disease, valvular disease, or other.

#### 2.2.4 Coronary disease

A history of coronary artery disease was noted if the patient had experienced a myocardial infarction, or had undergone treatment with percutaneous coronary intervention or aortocoronary bypass surgery.

### 2.3 Procedure for invasive electrophysiology and catheter ablation

Anti-arrhythmic drugs are routinely withdrawn at least five half-lives prior to CA in our department. However, confirmation of such withdrawal was not available for all patients. Conscious sedation was limited to minor doses of midazolam or fentanyl to avoid suppression of the PVCs. No patients were under general anesthesia during the CA procedure. If PVCs did not occur at baseline, isoprenaline infusion (2–10 µg/min) or pacing maneuvers were started.

AM or PM was performed on a case-by-case basis, with AM as the preferred method in patients with a sufficient number of PVCs, and PM as an alternative or supplementary method. An insufficient number of PVCs was defined as cases in which the operator had aborted the procedure due to an inability to identify the site of origin because an insufficient number of PVCs had occurred. Electroanatomical mapping was performed with either the CARTO-System (©Biosense Webster, Inc., Irvine, CA, USA) or the EnSite NavX system (©Abbott Medical, Lake County, IL, USA), at the discretion of the operator. The specific workflow of the procedure and the final decision to perform CA or not was made by the operator.

### 2.4 Statistical analysis

All data were analyzed in SPSS Statistics for Windows, version 26.0 (SPSS Inc., Chicago, IL, USA). All normally distributed continuous data were reported as means ± standard deviations, and group differences were evaluated with Student's *t*-test. All non-normally distributed continuous data were reported as the median and interquartile range (IQR), and group differences were evaluated using the Mann–Whitney *U*-test. All categorical variables were reported as numbers and percentages, and group differences were evaluated through the application of the Chi-square or Fisher's exact test, as appropriate. Receiver operating characteristic (ROC) analyses were used to analyze the discriminative power regarding the 24-h PVC burden and to find the burden that showed the optimal discriminatory value for sufficient PVCs during CA and the choice of mapping strategy. Statistical significance was defined as *p* < 0.05.

## 3 Results

### 3.1 Patient population

**Figure 1:**
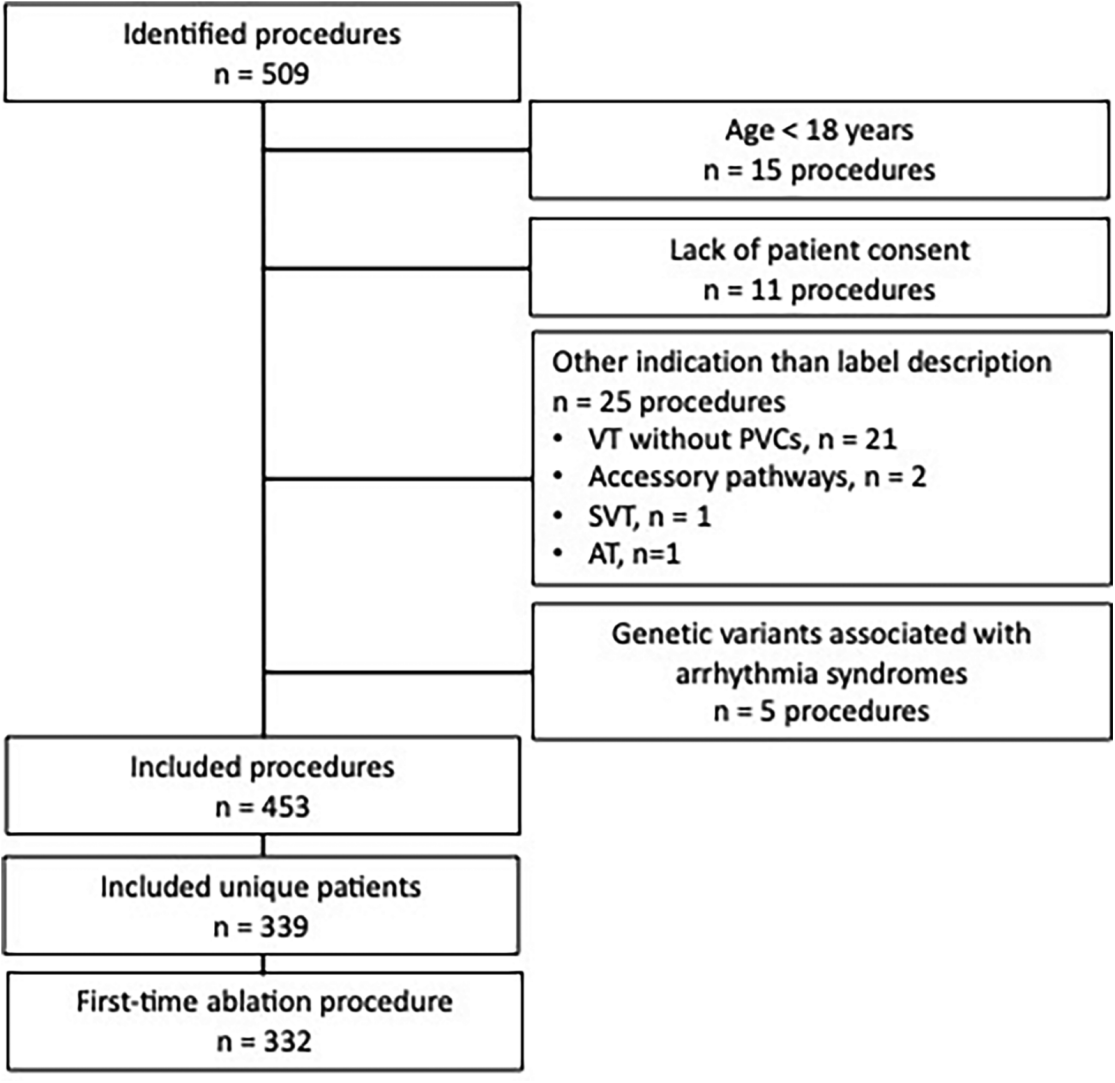
Flowchart for inclusion and exclusion of patients.

A total of 332 patients who had been referred for their first-time CA for PVCs were included (Figure 1). The cohort comprised 208 (63%) women and had a median age of 50 years (IQR 39–60) (Table 1). A history of cardiovascular risk factors or comorbidities was recorded in 117 patients (35%); hypertension was the most common and was recorded in 56 patients (17%). Anti-arrhythmic drugs, including beta-blockers and non-dihydropyridine calcium-channel antagonists, were used by 231 patients (70%); beta-blockers were the most used (201 patients, 61%).

**Table 1:** Clinical characteristics and results of preprocedural evaluation of patients admitted for catheter ablation of PVCs in regard to a sufficient number of PVCs during CA.

Clinical characteristics	All patients, *N* = 332	Procedure aborted due to insufficient PVCs, *n* = 46 (14%)	CA performed, *n* = 286 (86%)	*p*-value
Women, *n* (%)	208 (63)	28 (61)	180 (63)	0.8
Age, median, years (IQR)	50 (39–60)	48 (39–56)	50 (39–61)	0.3
Comorbidity, *n* (%)	117 (35)	14 (30)	103 (36)	0.5
Hypertension	56 (17)	8 (17)	48 (17)	0.9
Other arrhythmias^a^	23 (7)	3 (7)	20 (7)	1.0
Dyslipidemia	25 (8)	2 (4)	23 (8)	0.6
Coronary artery disease	24 (7)	1 (2)	23 (8)	0.2
Diabetes	15 (5)	2 (4)	13 (5)	1.0
Heart failure	13 (4)	1 (2)	12 (4)	1.0
Valve disease	14 (4)	2 (4)	12 (4)	1.0
Medication				
More than one AAD	29 (9)	4 (9)	25 (9)	1.0
Beta blocker	201 (61)	32 (70)	169 (59)	0.2
Calcium antagonist	15 (5)	3 (7)	12 (4)	0.5
Flecainide	15 (5)	1 (2)	14 (5)	0.7
Symptoms, *n* (%)	310 (93)	43 (94)	267 (93)	1.0
Palpitations	246 (74)	34 (74)	212 (74)	1.0
Other symptoms	99 (30)	15 (33)	84 (29)	0.7
Fatigue	61 (18)	12 (26)	49 (17)	0.1
Dyspnea	46 (14)	5 (11)	41 (14)	0.5
Presyncope	41 (12)	4 (9)	37 (13)	0.6
Syncope	33 (10)	6 (13)	27 (9)	0.4
ECG				
Heart rate, median (IQR)	73 (63–83)	68 (60–78)	73 (63–83)	0.2
QRS-width (ms), median (IQR)	140 (130–140)	140 (130–150)	140 (130–140)	0.3
PVC coupling interval (ms), median (IQR)	520 (460–580)	530 (435–620)	520 (460–580)	1.0
PVCs with inferior axis and LBBB pattern	290 (87)	40 (87)	250 (87)	0.9
24-h ECG				
No. of PVCs, median (IQR)	14,820 (6,000–26,000)	8,332 (3,066–20,974)	16,007 (6,509–26,205)	0.055
%PVCs, median (IQR)	17 (8–26)	12 (6–26)	17 (9–26)	0.3
Other examinations				
Echocardiography normal, *n* (%)^b^	238 (73)	37 (80)	201 (72)	0.2
EF > 50%, *n* (%)	291 (90)	42 (93)	249 (90)	0.6
MRI normal, *n* (%)^c^	88/122 (72)	16/21 (76)	72/101 (71)	0.6
Exercise test with PVCs, *n* (%)^d^	190 (91)	22 (79)	168 (93)	**0** **.** **015**

PVC, premature ventricular complexes, n, number of procedures, IQR, interquartile range, AAD, anti-arrhythmic drug, EF, ejection fraction, MRI, magnetic resonance imaging.

Continuous data were analyzed with *t*-test (*) or Mann–Whitney *U-*test. Categorical variables were evaluated with Chi-square or Fisher’s exact test, as appropriate. The *p*-values are for insufficient or sufficient PVCs.

^a^ Other arrhythmias comprise atrial fibrillation (AF) (*n* = 12), atrioventricular nodal reentry tachycardia (AVNRT) (*n* = 5), atrial tachycardia (*n* = 3), AV-block (*n* = 2), and nodal rhythm (*n* = 1).

^b^ Normal echocardiography as defined by the operator.

^c^ Normal MR as defined by the operator.

^d^ PVCs at any time point prior to, during, or after an exercise test.

Bold indicate statistically significant values.

A total of 310 (93%) patients had PVC-associated symptoms. Palpitations were the most common of these, reported by 246 patients (74% of all patients) (Table 1). The median number of PVCs in the 24-h ECG recording was 14,820 (IQR 6,000–26,000), and the median PVC burden was 17% (IQR 8–26%). The most frequent PVC configuration was the inferior axis and left bundle branch block (I-LBBB), which was present in 290 patients (87% of all patients).

The majority of the patients had a structurally normal heart, as identified by echocardiography [normal in 238 of 325 patients (73%)] and/or MRI [normal in 88 of 122 patients (72%)]. The most common finding in echocardiography and MRI showed a dilated left ventricle with or without reduced EF.

### 3.2 Associations between clinical parameters and a sufficient number of PVCs during CA

**Figure 2:**
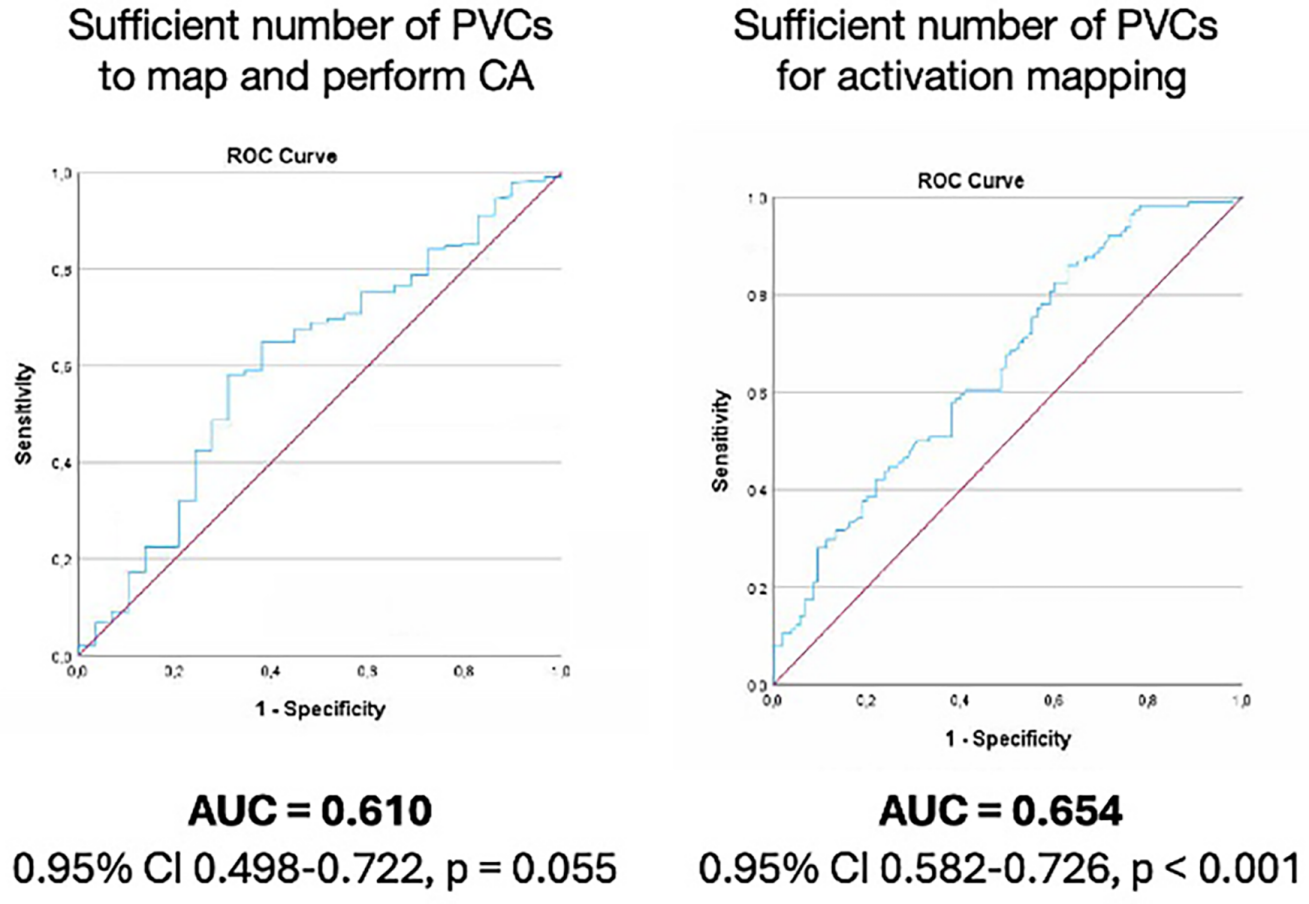
Number of PVCs in 24 h ECG for sufficient number of PVCs during catheter ablation procedure (left) and for activation mapping as sole mapping strategy (right). PVC, premature ventricular complexes; ROC, receiver operating characteristic analysis.

In our cohort, the CA procedure was aborted due to an insufficient number of PVCs in 46 patients (14%). We compared clinical parameters in these patients to those in patients with a sufficient number of PVCs for CA to be performed (Table 1). Patients with a sufficient number of PVCs had nominally more PVCs during the 24-h ECG recording than did patients with an insufficient number of PVCs, with a strong statistical trend [16,007 (IQR 6,509–26,205)] vs. [8,332 (IQR 3,066–20,974), *p* = 0.055]. Based on this and the findings of previous studies (12), we analyzed this association further. In the ROC analysis of the number of PVCs in the last 24-h ECG and sufficient PVCs during CA, the area under the curve (AUC) was 0.610 (95% CI 0.498–0.722, *p* = 0.055) (Figure 2). The cut-off number of PVCs with the best predictive values was 10,000 PVCs per 24 h, with a positive predictive value (PPV) of 67% and a negative predictive value (NPV) of 57%, respectively.

The median number of days from the 24-h ECG recording to CA in our cohort was 179 (IQR 100–289). Since the number of PVCs per 24 h fluctuates significantly over time (16–18), we analyzed the influence of the period of time from the recording to CA. We compared the ROC curve of 24-h ECGs recorded >6 months prior to CA and <6 months prior to CA. The AUCs for these ROC curves were not statistically different (0.584 vs. 0.665, *p* = 0.4).

The only other parameter that showed a statistically significant difference between patients with sufficient and insufficient number of PVCs during CA was the presence of PVCs observed in relation to exercise testing (Table 1). It is important to note that in our study this included PVCs at any time during the exercise test procedure, i.e., at rest during the preparation for testing, during exercise, or during recovery. PVCs during the exercise testing were reported in 190 patients (91% of patients with an exercise test, 190/209). PVCs in relation to an exercise test were reported more often in patients with sufficient than in patients with insufficient PVCs during CA (93 vs. 79%, *p* = 0.015). The PPV and NPV for PVCs related to exercise testing for sufficient number of PVCs during the CA procedure were 88% and 32%, respectively.

### 3.3 Associations between clinical parameters and mapping strategy

Electroanatomical mapping was performed in 245 patients (74% of all patients) (Table 2). AM was performed in 141 patients (58%), and PM was used either alone or in addition to AM in 104 patients (42%). The remaining patients did not proceed with electroanatomical mapping during their CA due to an insufficient number of PVCs (14% of all patients), only electrophysiologic examination (11% of all patients), or complications/patient withdrawal during the procedure (1% of all patients). In CA procedures in which PM was performed, either by itself or with AM, 73% of the patients were female compared with 57% in procedures in which AM was used as the only strategy (*p* = 0.012). The PVC coupling interval was longer in patients for whom AM could be used alone [535 (480–580) vs. 500 (440–580), *p* = 0.023].

**Table 2:** Clinical characteristics and results from the preprocedural evaluation of patients admitted for CA of PVCs, in regard to activation as a mapping strategy.

Clinical characteristics	Activation mapping, *n* = 141 (58%)	Pace mapping ± activation mapping, *n* = 104 (42%)	*p*-value
Women, *n* (%)	81 (57)	76 (73)	**0** **.** **012**
Age (years), median (IQR)	50 (38–62)	50 (38–59)	0.7
Comorbidity, *n* (%)	54 (38)	31 (30)	0.2
Hypertension	25 (18)	14 (14)	0.4
Other arrhythmias	11 (8)	6 (6)	0.5
Dyslipidemia	8 (6)	11 (11)	0.2
Coronary artery disease	12 (9)	7 (7)	0.6
Diabetes	6 (4)	4 (4)	1.0
Heart failure	11 (8)	2 (2)	**0.047**
Valve disease	7 (5)	5 (5)	1.0
Medication			
More than one AAD	10 (7)	8 (8)	0.9
Beta blocker	84 (60)	57 (55)	0.5
Calcium antagonist	5 (4)	5 (5)	0.6
Flecainide	4 (3)	7 (7)	0.2
Symptoms	127 (90)	99 (95)	0.1
Palpitations	101 (72)	79 (76)	0.4
Other symptoms	28 (20)	38 (37)	**0** **.** **004**
Fatigue	29 (21)	17 (16)	0.4
Dyspnea	21 (15)	15 (14)	0.9
Presyncope	17 (12)	10 (10)	0.5
Syncope	12 (9)	9 (9)	1.0
ECG			
Heart rate, median (IQR)	70 (61–79)	73 (64–83)	0.4
QRS-width (ms), median (IQR)	140 (130–140)	140 (130–150)	0.2
PVC coupling interval (ms), median (IQR)	535 (480–580)	500 (440–580)	**0** **.** **023**
PVCs with inferior axis and LBBB pattern	117 (83)	95 (91)	0.06
24-h ECG			
No. of PVCs, median (IQR)	19,769 (10,564–30,526)	15,237 (6,000–25,033)	**0** **.** **022**
%PVCs, median (IQR)	20 (10–29)	18 (10–26)	0.2
Other examinations			
Echocardiography normal, *n* (%)	94 (68)	73 (72)	0.4
EF > 50%, *n* (%)	120 (88)	92 (91)	0.4
MRI normal, *n* (%)	33/49 (67)	23/33 (70)	0.8
Exercise test with PVCs, *n* (%)	88 (96)	59 (92)	0.5

PVC, premature ventricular complexes, *n*, number of procedures, IQR, interquartile range, AAD, anti-arrhythmic drug, ECHO, echocardiogram, EF, ejection fraction, MRI, magnetic resonance imaging.

Bold indicate statistically significant values.

Continuous data were analyzed with *t*-test (*) or Mann–Whitney *U-*test. Categorical variables were evaluated with Chi-square or Fisher’s exact test, as appropriate. The *p*-values are for insufficient no. of PVCs or sufficient no. of PVCs.

Patients who were treated for PVCs with CA based on AM alone had more PVCs in the 24-h ECG [19,769 (10,564–30,526) vs. 15,237 (6,000–25,033), *p* = 0.022]. The ROC curve for AM as the sole mapping strategy and the number of PVCs in the 24-h ECG prior to CA had an AUC value of 0.654 (95% CI 0.582–0.726, *p* < 0.001). Those with more than 10,000 PVCs in the 24-h ECG had PPV and NPV for mapping by AM alone of 75% and 41%, respectively.

In our cohort, CA that was guided by AM alone was not associated with a higher success rate (82 vs. 74%, *p* = 0.2). However, fewer patients who were treated with CA based on AM alone compared with those who were treated with PM required a redo procedure (15 vs. 26%, *p* = 0.031). There was no difference in the procedure time between CA procedures that were guided by AM alone and those in which PM was used either alone or in addition to AM [203 min (IQR 163–275) vs. 231 min (IQR 170–268), *p* = 0.3].

## 4 Discussion

We collected data retrospectively from adult patients who had been admitted for CA of PVCs. The objective was to quantify the association between common clinical parameters and a sufficient number of PVCs and between mapping strategy and a sufficient number of PVCs during the procedure in a representative real-world cohort. Patients who had an insufficient number of PVCs to proceed with any electroanatomical mapping also had fewer PVCs in the last recorded 24-h ECG. Patients with an insufficient number of PVCs at the time of the procedure were also less likely to have had PVCs in relation to exercise testing. Furthermore, patients for whom AM alone could guide CA had more PVCs in the 24-h ECG, had longer coupling intervals in their PVCs, and more of them were male. In our cohort, mapping strategy was not associated with success rate or procedure time.

### 4.1 Prediction of a sufficient number of PVCs during catheter ablation

An insufficient number of PVCs to perform electroanatomical mapping sufficient for CA is a well-known unresolved problem that faces electrophysiologists frequently (9, 10). The use of various protocols to provoke PVCs in patients in whom they do not occur spontaneously has been suggested, but the response cannot be predicted with any established methods (10, 17, 19). Provocation protocols were also used in our cohort, but 14% of patients still had an insufficient number of PVCs to proceed with electroanatomical mapping. These data illustrate the need for predictors in the preprocedural assessment and selection of patients. The few studies that have focused on the insufficient number of PVCs during CA have not identified any parameters that show robust predictive values (11, 12). In one study that was focused on preprocedural PVC burden and outcome of CA procedures, no correlation was found, and the researchers concluded that patients with clear symptoms should be admitted for CA even with a low burden of PVCs, i.e., should not be excluded from attempts at CA (11). In contrast, Demir et al. found an association between 24-h PVC burden and a sufficient number of PVCs during CA and suggested a 20% PVC burden as a cut-off for optimal CA outcomes (12). Our data suggest that PVC burden in the last 24-h ECG, recorded at a median of 6 months (179 days) from 24-h ECG to CA, has a moderate predictive value (AUC = 0.610) that there will be sufficient PVCs to proceed with electroanatomical mapping during CA. We found that >10,000 PVCs in the 24-h ECG had a PPV of 67% and a NPV of only 57%.

Furthermore, our data indicate that a higher PVC burden in the 24-h ECG increases the probability that AM can be used as the sole mapping strategy. This is relevant since AM is generally considered more accurate than PM to guide the CA of PVCs, although few studies have compared the two mapping strategies. PM might successfully guide CA procedures when AM is not feasible due to a low number of PVCs during the procedure (9, 20), and, in direct comparisons, the acute and 1-month success rates are similar for AM and PM (20). In our cohort, neither the success rate nor the procedure time differed for the two mapping strategies.

The patients in our cohort underwent CA without general anesthesia, which is the standard and most common procedure for CA of PVCs in our hospital. A previous study has shown that AM is more commonly used as the sole mapping method during local anesthesia compared to general anesthesia, and higher acute success rate with local anesthesia, independent of the use of AM (21). This may also have affected the results in our cohort, but the dataset did not allow for further analysis of the association between sedation and the number of PVCs during CA.

Our data also identified PVCs related to exercise testing as the only other parameter that was associated with a sufficient number of PVCs at the time of the procedure. However, the available patient records did not allow us systematically to associate specific relationships between exercise and PVCs (before, during, or after) with the occurrence of PVCs during CA. This should be further explored in prospective studies.

Overall, our data illustrate a need to continue work to identify preprocedural parameters or sets of parameters that can be used to predict the presence of sufficient PVCs during CA to enable completion of the procedure and to develop personalized protocols to elicit PVCs in patients in whom they do not occur spontaneously.

### 4.2 Special considerations to increase the predictive value of 24 h ECGs

We included in our study the last available 24-h ECG recordings, regardless of the time that had elapsed between the recording and CA. Information regarding the use of anti-arrhythmic drugs during the recording was not available. In our center, the waiting lists for CA of PVCs during the study period were at times long, which is reflected in the time from referral to CA. The median number of days from the 24-h ECG to CA was 179, with considerable variation (IQR 100–289). Whether the time from the last 24-h ECG recording to the CA procedure influences the predictive value has not been explicitly tested in previous studies. Based on the well-known considerable variation in PVC burden over time, we expected that the time from the 24-h ECG to CA would influence the predictive value. However, we did not find a significant difference in the predictive value for sufficient PVCs during CA in cases in which 24-h ECGs had been recorded more than or less than 6 months before the procedure. Nevertheless, the predictive value of the 24-h ECG recording at different time points prior to CA requires further investigation, as does testing of the predictive value of other parameters that can be derived from a 24-h ECG, such as diurnal variation and relationship to heart rate (11).

### 4.3 Limitations

Our study provides real-world data from a representative, high-volume center, but the retrospective data collection creates an inherent limitation due to potential unsystematic changes in clinical practice during the study period and the variable accuracy and practice of reporting in patient records. This was most notable regarding our data on the use of anti-arrhythmic drugs at different time points and information from exercise tests that did not allow analysis of the specific relationship between different patterns of PVC-exercise relationships and PVC occurrence during CA.

## 5 Conclusion

In our retrospective analysis of real-world data from a representative cohort, the number of PVCs in the 24-h ECG that had been performed closest to the CA procedure showed moderate predictive values for a sufficient number of PVCs at the time of the CA, and for the suitability of the use of CA guided by AM alone. The number of PVCs in relation to exercise testing was associated with a sufficient number of PVCs during CA to perform electroanatomical mapping. Prospective studies to identify other predictive parameters that can be used to guide the selection of patients for the cost-effective use of CA for PVCs, and guide shared decision-making, are needed.

## Data Availability

The availability of the datasets presented in this article limited by regulations in Norway. The data supporting the conclusions of this article will be made available by the authors, according to the formal regulations in Norway. Requests to access the datasets should be directed to Mathis Korseberg Stokke, m.k.stokke@medisin.uio.no.
